# Army Medical Society, at Jackson, Tenn.

**Published:** 1863

**Authors:** J. R. Pearce

**Affiliations:** Sec’y.; Jackson, Tenn.


### ARMY MEDICAL SOCIETY, AT JACKSON, TENN.

JACKSON, TENN., *April* 27, 1863.

EDITOR EXAMINER:—Preliminary to the organization of a Medical Society here, on the evening of April 2d a number of medical officers convened at the office of the General Hospital.

On motion of Dr. H. W. DAVIS, Dr. S. YORK was elected Temporary-President, and Dr. J. R. PEARCE, Secretary.

The president, on taking the chair, stated more fully the object of the meeting,—to be the formation of a Medical Society to consist of the medical officers of the 16th Army Corps. That it is true of medicine as anything else; that in union there is strength; and that no physician is worthy of his noble profession, who is not ever ready to do all in his power to elevate it to greater and still greater usefulness; that the surgeons in and near Jackson may spend an evening or two of each week in medical association, not only with profit and pleasure to themselves, but with great advantage to the sick and afflicted soldiers, whose sufferings they are so often called to alleviate, &c.

Drs. HUNT, BRIDGES, and MILLS were appointed a committee to draft a Constitution and By-laws, and report at the next meeting.

The Society met Saturday evening, April 4th, and adopted the Constitution and By-laws as reported. Dr. S. YORK was elected President; Dr. C. A. HUNT, Vice-President; Dr. J. R. PEARCE, Secretary.

On signing the Constitution and By-laws, all present were considered members, viz.: S. York, Surgeon, 54th Ill. Infantry; C. A. Hunt, Surgeon, 126th Ill. Infantry; Henry W. Davis, Surgeon, 18th Ill. Infantry; Acting Chief of Hospitals, J. W. Cameron, Surgeon 62d Ill. Infantry; R. F. Stratton, Surgeon, 11th Ill. Cavalry; H. E. Foote, Surgeon, 22d Ohio Infantry; E. W. Mills, Assistant-Surgeon, 126th Ill. Infantry; E. A. Lee, Assistant, 54th Ill. Infantry; T. D. Washburn, Assistant, 126th Ill.; V. R. Bridges, Assistant-Surgeon, 62d Ill. Infantry; Dr. Buffon, 1st Sergeant in Co. K. 54th Ill.; S. R. Mitchell, H. T. Garnett, J. R. Pearce, Contract-Surgeons in General Hospital; L. C. Halsted, Assistant-Surgeon, 7th Wis. Battery; Lieut.-Col. Allen, Medical Inspector U. S. A., being present, was elected Honorary Member.

The Society meets every Saturday evening. Discussions are held upon the various diseases incident to camp; and an essay is read, by some member previously appointed, and discussed. It is, constitutionally, incumbent upon each member to report any surgical case of interest he may be called upon to treat.

At the meeting on 18th inst., the accompanying essay was read by C. A. HUNT, Surgeon, 126th Ill., and ordered by the Society to be forwarded to *The Chicago Medical Examiner* for publication, together with the proceedings of the early organization of the Society.

J. R. PEARCE, *Sec'y.*

